# Significant of adiponectin in gastropathy: Case-controlled study

**DOI:** 10.1016/j.amsu.2019.09.008

**Published:** 2019-09-19

**Authors:** Batool Mutar Mahdi

**Affiliations:** Head of HLA Research Unit, Department of Microbiology, Al-Kindy College of Medicine, University of Baghdad, Iraq

**Keywords:** Gastropathy, Adiponectin, Obesity

## Abstract

**Background:**

Gastropathy is a medical broad term applied for stomach diseases that affect mucosal lining characterized by epithelial injury. There are many types of gastropathy ranging from harmless conditions to more serious ones like cancer.

**Aim of the study:**

to assess the significant effect of adiponectin and its level in patients with gastropathy.

**Patients and methods:**

This case-control study includes 35 patients with gastropathy confirmed by gastroscope and thirty control group. Anthropometric Measurements like Weight, height, waist circumference, body mass index, and waist circumference. Blood sample was collected from the patients and control group and serum tested for lipid profile that includes (cholesterol, triglyceride, HDLP, LDLP) (Human-Germany), and adiponectin (Shanghai Biologic Technology-China) using Enzyme-Linked Immunosorbent Assay (ELISA).

**Results:**

There is a significant difference between the two groups regarding height, weight, waist circumference, and waist to hip ratio (P-value 0.002,0.009, 0.002 and 0.015 respectively). Regarding adiponectin, there is no significant difference between patients (9.54 ± 5.821 and control 9.119 ± 7.062)(P = 0.796). Lipid profile showed a significant increase in cholesterol (p = 0.0001) and triglyceride (0.007) in patients group while there is no significant difference in HDL, LDL and LDL/HDL ratio.

**Conclusions:**

Adiponectin had no role in the development of gastropathy. Patients with gastropathy had a significant increase in the serum level of cholesterol and triglyceride. There is a significant negative correlation between adiponectin and weight, body mass index, waist circumference, triglyceride, and low-density lipoprotein.

## Highlights

• Gastropathy is a medical broad term applied for stomach diseases.
• Effect of adiponectin and its level in patients with gastropathy.
• This case-control study includes 35 patients with gastropathy confirmed by gastroscope and thirty control group.
• Anthropometric Measurements and blood sample were collected from two groups.
• There is a significant difference between two groups regarding height, weight, waist circumference and waist to hip ratio while adiponectin there is no significant difference between two groups.

## 1 Introduction

Gastropathy is a medical broad term applied for stomach diseases that affect mucosal lining characterized by epithelial injury. Patients usually complain from different symptoms according to the cause like *Helicobacter pylori* infection [1]. Diagnosis can be achieved by the collaboration of clinician, endoscopist, and histopathologist [2]. There are many conditions that lead to gastropathy like gastritis, gastroenteritis, and peptic ulcer [3]. It can be classified according to the duration of disease development, inflammatory type, and etiology. One of the causes of gastropathy and gastric erosion is obesity. In obese individuals, visceral fat plays an important role in metabolic disorders which is due to Dysregulation of bioactive molecules like adiponectin that secreted from visceral fat [4]. Adipose tissue and adipocytes were synthesized adiponectin which had multifunctional anti-inflammatory properties. Adiponectin is an adipose tissue-derived cytokine that has an important role in the inflammation [5]. It circulates throughout the body and its plasma concentrations are decreased in obese individuals [6]. The anti-inflammatory properties of adiponectin have a protective role in many gastrointestinal diseases [7]. Adiponectin inhibits chemokine production and inflammatory responses like inhibiting macrophage infiltration and release of proinflammatory cytokines [8]. It associated with increased apoptotic cells and decreased expression of prostaglandin E2 and cyclooxygenase-2 [9]. On the other hand, adiponectin is one of the members of the adipokine family that had a role in many diseases [10]. The relation between adiponectin and gastric erosion showed low serum adiponectin which is an independent risk factor for gastric erosion [11].

Thus, adiponectin has a protective role against gastric mucosal injury. Because the incidence of gastropathy is increased in obese individuals, one can hypothesize that lower serum adiponectin level in obese subjects is a risk factor for gastropathy.

The main aim of this study is to assess and clarify the significant role, effect of adiponectin and its level in patients with gastropathy. This study was to elucidate the correlation between adiponectin and different parameters like weight, waist circumference, and lipid profile.

## 2 Patients and methods

This case-control study includes 35 patients with gastropathy confirmed by gastroscope (gastroscopy: GIF-H260; Olympus, Tokyo, Japan, and Display screen; Olympus OEV-261H liquid crystal display monitor; Olympus, Tokyo, Japan) in Gastroenterology Unit in Al-Kindy Teaching Hospital in Baghdad-Iraq from January 2017 to May 2018 and the other group was thirty individuals as control.

• The inclusion criteria were:

Patients complaining from dyspepsia, upper abdominal discomfort, acid regurgitation, and heartburn.

• The exclusion Criteria were:

Patients with peptic ulcer or cancer and patients on medication like proton pump inhibitors and non steroidal anti-inflammatory drugs.

The study was accepted by the scientific ethical committee of Al-Kindy College of medicine and Al-Kindy Teaching Hospital. Informed consents and agreement were obtained from patients.

Anthropometric Measurements like Weight in kilograms, height in centimeters, waist circumference in centimeters, body mass index (weight in kilograms divided by the square of height in meters) and waist circumference that measured in centimeters at the end of normal expiration halfway between the lowest rib and the iliac crest with the examiner standing at the side to ensure that the measuring tape is horizontal across the back and the front of the participant [12].

Blood samples were collected from the patients and control group and serum tested for lipid profile that includes (cholesterol, triglyceride, HDLP, LDLP) (Human-Germany), and adiponectin (Shanghai Biologic Technology-China) using Enzyme-Linked Immunosorbent Assay (ELISA).

The work is fully compliant with the STROCSS criteria (www.strocssguideline.com). The completed STROCSS checklist stating the page numbers was submitted. The work has been reported in line with the STROCSS criteria [13]. The work with Thai clinical trial registry ID is TCTR20190820001.

**Statistical analysis:** done using MiniTab version 3.0 software. Results were analyzed using a Chi^2^ test for frequencies, student t-test for means and standard Error Mean and correlation coefficient by Pearson correlation. A p-value of less than 0.05 was considered statistically significant.

## 3 Results

The patient's group sex and age were not a significant difference with the control group. There is a significant difference between two groups regarding height, weight, waist circumference, and waist to hip ratio (P-value 0.002,0.009,0.002 and 0.015 respectively). About adiponectin, there is no significant difference between patients (9.54 ± 5.821 and control 9.119 ± 7.062)(P = 0.796). Lipid profile showed a significant increase in cholesterol (p = 0.0001) and triglyceride (0.007) in patients group while there is no significant difference in HDL, LDL, and LDL/HDL ratio. Regarding Pearson correlation analysis demonstrated a negative correlation between adiponectin and age, height, weight, BMI, waist circumference, waist to hip ratio and lipid profile, while LDL/HDL showed positive correlation as shown in table-1.

**Table 1:** Difference of various parameters between patients with gastropathy and control group with Pearson correlation analysis of adiponectin.

Parameters	Patients with gastropathy No.=35 X±SD	Control group No.=30 X±SD	p- value	Pearson correlation (r) (P-value)
Age (years)	44.3±17.6 (23-57)	38.2±12.4 (24-60)	0.10	- 0.05 (0.77)
Males %	15 (42.85)	16 (53.33)	0.39	
Females %	20 ( 57.14)	14 (46.66)		
Height (CM)	164.8±0.1	172.3±0.08	0.002	-0.13 ( 0.45 )
Weight (KG)	72.9±18.9	84.9±17.1	0.009	-0.37 ( 0.02 )
BMI (Kg/m^2^)	26.5±6.15	24.59±5.08	0.18	-0.32 (0.05)
Waist Circumference (Cm)	93.3±14.7	105.3±15.3	0.002	-0.32 (0.05)
Waist to hip ratio	1.04±17.0	1.13±10.9	0.015	-0.19 (0.26)
Adiponectin mg/ml	9.54±5.82	9.11±7.06	0.796	
Cholesterol (mg/ml)	266±1.02	155.2±1.54	0.0001	-0.09 (0.60)
Triglyceride (mg/ml)	301±226	174.2±97.4	0.007	-0.32 (0.05)
HDL (mg/ml)	45.3±16.1	50.0±14.8	0.227	- 0.08 (0.61)
LDL (mg/ml)	170.2±92.8	164±104	0.811	- 0.38 (0.02)
LDL/HDL	4.1±2.7	2.94±1.65	0.29	+0.19 (0.26)

## 4 Discussion

Most of the patients were complaining from Gastropathy and one of its causes is obesity because one of the complications of the obesity in addition to diabetes mellitus and cardiovascular diseases is gastrointestinal and gastropathy [14]. Adiponectin derived and secreted from adipose tissues and had an anti-inflammatory and protective role against inflammation and malignancy [15].

The findings of this study showed that there was no significant difference in the level of adiponectin between patients group and control one and there is a negative correlation between adiponectin and different parameters that examined in those patients. There is a significant difference between the two groups regarding weight, waist circumference, cholesterol, and triglyceride. Adiponectin which had anti-inflammatory property showed a decreased level in obese patients independent on age and sex [16].

Comparing with another study that reported reduced serum concentration of adiponectin in gastric, esophageal and colorectal cancer [17]. There is also a lack of any correlation between adiponectin receptors expression and stage of the gastric tumor [18]. In addition to that reduction in serum, adiponectin refers to adipose tissue wasting [19]. An additional study showed decreased serum adiponectin in patients with erosive gastritis independent on BMI [20]. At the same time, Kim HJ et al., 2007, reported that BMI had an association with gastric ulcer and duodenal ulcer and GERD [21].

Discussing and analyzing different factors like age, sex, alcohol intake, smoking, BMI, lipid profile like cholesterol, triglyceride, glucose, and insulin in 2400 patients, it showed that BMI was significantly higher while adiponectin level was significantly lower in gastritis patients than in non-gastritis group. This means a possible role of hypoadiponectinemia in erosive gastritis and also in gastric cancer [22] while in our study showed no significant difference in the level of adiponectin with different parameters. Adiponectin showed down-regulation of inflammation both in vitro and in vivo in many gastrointestinal disorders like autoimmune disease, inflammation, malignant and in many sites of tract [23]. In addition to that adiponectin had an association with other diseases like diabetes mellitus [[24], [25], [26], [27]] and polycystic ovary [28]. Adiponectin mediates energy metabolism and functions as an immunomodulator [29]. Circulating adiponectin is involved in the pathogenesis of GERD and may predispose to Barrett's esophagus [30].

Limitations of this study were sample size, characteristics of patients' selection, study design and loss of follow –up to patients.

## 7 Conclusions

Adiponectin had no role in the development of gastropathy. Patients with gastropathy had a significant increase in the serum level of cholesterol and triglyceride. There is a significant negative correlation between adiponectin and weight, body mass index, waist circumference, triglyceride, and low-density lipoprotein.

## Ethical approval

Al-Kindy college of medicine committee.

## Sources of funding

Non.

## Author contribution

Dr Batool mutar mahdi gave the proposal, wrote the paper and statistics.

## Conflicts of interest

None.

## Research registration number

Name of the registry: Thai clinical trial registry.

The work with Thai clinical trial registry ID is TCTR20190820001.

Hyperlink to the registration (must be publicly accessible): http://www.clinicaltrials.in.th/index.php?tp=regtrials&menu=trialsearch&smenu=fulltext&task=search&task2=view1&id=5136.

## Guarantor

Ass. Prof Dr. Riyadh Mohamad Hasan.

Lecturer Huda Saleem Hantosh.

## Funding

None.

## Provence and peer review

Not commissioned, externally peer reviewed.
